# Role of Endocrine-Disrupting Engineered Nanomaterials in the Pathogenesis of Type 2 Diabetes Mellitus

**DOI:** 10.3389/fendo.2018.00704

**Published:** 2018-11-26

**Authors:** Ayushi Priyam, Pushplata Prasad Singh, Shweta Gehlout

**Affiliations:** TERI Deakin Nanobiotechnology Centre, The Energy and Resources Institute, New Delhi, India

**Keywords:** engineered nanomaterial (ENM), type 2 diabetes mellitus (T2DM), endocrine disruptor, insulin resistance, reduced insulin sensitivity, oxidative stress, *in vitro* and *in vivo* studies, epidemiological evidences

## Abstract

Nanotechnology has enabled the development of innovative technologies and products for several industrial sectors. Their unique physicochemical and size-dependent properties make the engineered nanomaterials (ENMs) superior for devising solutions for various research and development sectors, which are otherwise unachievable by their bulk forms. However, the remarkable advantages mediated by ENMs and their applications have also raised concerns regarding their possible toxicological impacts on human health. The actual issue stems from the absence of systematic data on ENM exposure-mediated health hazards. In this direction, a comprehensive exploration on the health-related consequences, especially with respect to endocrine disruption-related metabolic disorders, is largely lacking. The reasons for the rapid increase in diabetes and obesity in the modern world remain largely unclear, and epidemiological studies indicate that the increased presence of endocrine disrupting chemicals (EDCs) in the environment may influence the incidence of metabolic diseases. Functional similarities, such as mimicking natural hormonal actions, have been observed between the endocrine-disrupting chemicals (EDCs) and ENMs, which supports the view that different types of NMs may be capable of altering the physiological activity of the endocrine system. Disruption of the endocrine system leads to hormonal imbalance, which may influence the development and pathogenesis of metabolic disorders, particularly type 2 diabetes mellitus (T2DM). Evidence from many *in vitro, in vivo* and epidemiological studies, suggests that ENMs generally exert deleterious effects on the molecular/hormonal pathways and the organ systems involved in the pathogenesis of T2DM. However, the available data from several such studies are not congruent, especially because of discrepancies in study design, and therefore need to be carefully examined before drawing meaningful inferences. In this review, we discuss the outcomes of ENM exposure in correlation with the development of T2DM. In particular, the review focuses on the following sub-topics: (1) an overview of the sources of human exposure to NMs, (2) systems involved in the uptake of ENMs into human body, (3) endocrine disrupting engineered nanomaterials (EDENMs) and mechanisms underlying the pathogenesis of T2DM, (4) evidence of the role of EDENMs in the pathogenesis of T2DM from *in vitro, in vivo* and epidemiological studies, and (5) conclusions and perspectives.

## Background

Materials acquire unique characteristics when the size of the particle is reduced to nanoscale. Nanomaterials (NMs) are a universal set of nanoscale materials having at least one of the dimensions in the nano-range. With having at least one dimension in nanoscale as a common feature, nanoparticles, nanowires, nanosheets, nanotubes, and nanoplates can be stated as the key subsets of NMs (1). The various properties of a nanomaterial (NM), including its melting point, electrical conductivity, magnetic permeability, chemical reactivity and fluorescence, are determined by the particle size (2). Size-reduction of a material to nanoscale enhances its functional aspects and associated technological benefits. Therefore, the use of engineered nanomaterials (ENMs) in the development of advanced technologies for medicine, engineering and natural sciences has significantly increased since the start of the twenty-first century (3). ENMs are being incorporated into our everyday routine as a part of clothing, food, cosmetics, medicines, electronic goods, etc. However, in parallel to the technological advancements, the biosafety issues related to ENMs have also become a matter of apprehension. Whereas, for applications in medicine, ENMs are optimized to enhance their cellular uptake and/or targeting to the desired tissue, an inadvertent exposure to workers may raise health concerns (4, 5). Multiple studies have suggested that unlike their bulk counterparts, the ENMs are highly toxic and may lead to serious human and ecological health risks (6–8). The toxic outcomes of ENM exposure are largely accredited to their small size and increased chemical reactivity, which enhances their permeability to the target tissues which are otherwise not penetrated by larger but chemically identical materials (9). Noticeably, evidence from several research studies indicates functional similarities between the endocrine-disrupting chemicals (EDCs) and ENMs, which supports the view that different types of NMs may be capable of altering the physiological activity of the endocrine system (10–14).

**Figure 1:**
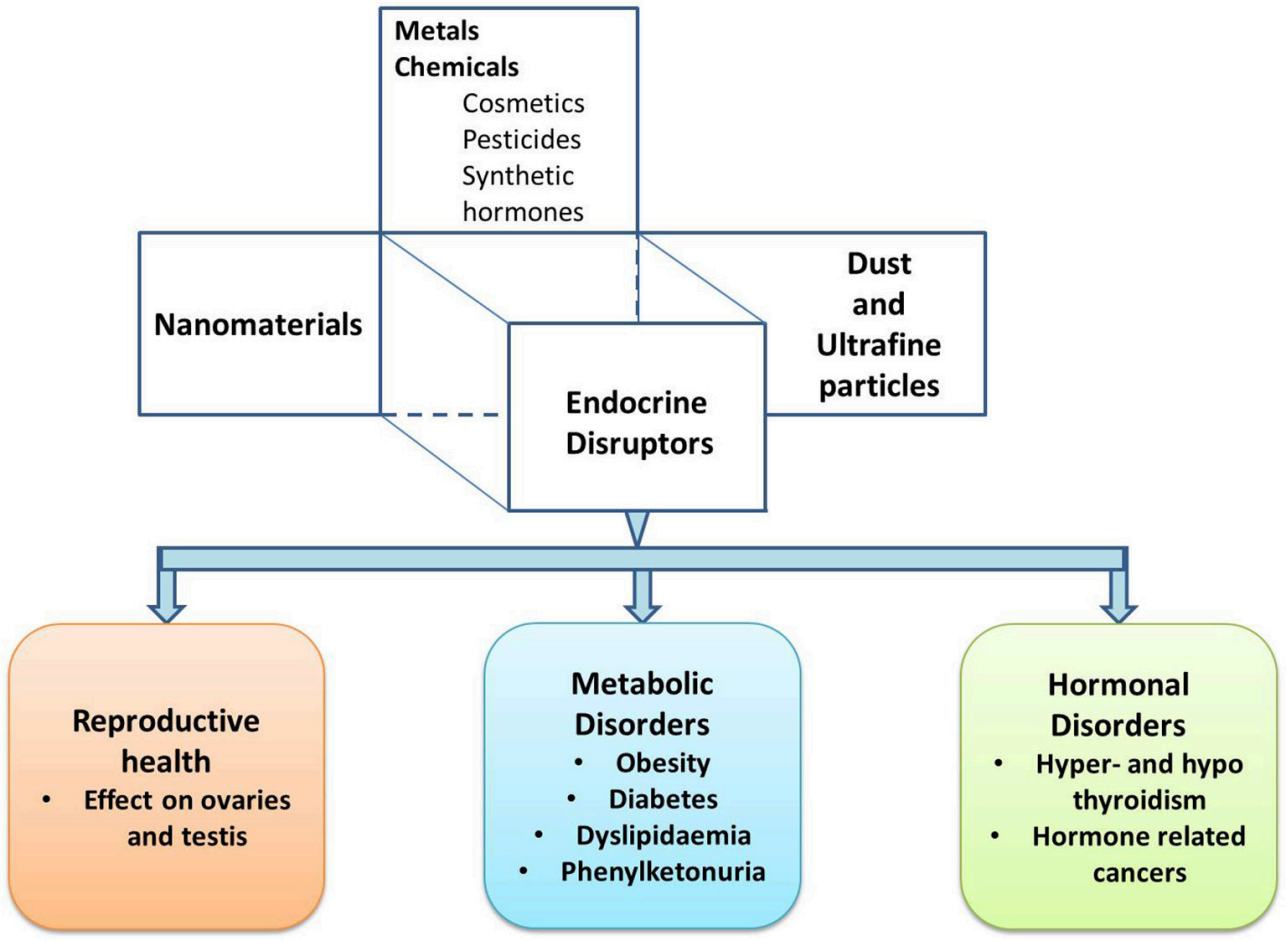
A schematic describing different types of endocrine disruptors and the associated endocrine disorders.

The WHO (World Health Organization) International Programme on Chemical Safety (IPCS) conducts research to understand the basis for the management of chemicals and related risks. According to the IPCS, “a potential endocrine disruptor is an exogenous substance or a mixture, possessing properties that can lead to endocrine disruption in an intact organism, or its progeny, or (sub) populations” (15). Further to add, the EDCs are elements present in our environment, food, and several consumer products that can interfere with synthesis, secretion, transport, metabolism, binding actions and elimination, and mimic the natural hormones. Consequently, this may lead to a deviation from the normal physiological function of the endocrine system to endocrine disruption. The EDCs and endocrine disrupting ENMs (EDENMs) are prevalent in various consumer goods such as agricultural chemicals, notably fertilizers and pesticides (16, 17), therapeutics (18), cosmetics (19, 20), and paints (19). There is accumulating evidence suggesting an increased presence of EDCs and ENMs in the environment, which putatively affects the functioning of the endocrine system, metabolic system and reproductive system (Figure 1). Hence, though the ENMs promise remarkable benefits, their successful application requires investigation of their impact on human health.

Both EDCs and NMs are regularly investigated for adverse impacts on health. Studies conducted to evaluate the effects of EDCs on health have generally reported deleterious outcomes like altered reproductive function in males and females (21–25), increased incidence of breast cancer (26–30), abnormal growth patterns (31–33), neurodevelopmental delays in children (34–36), and changes in immune functions (37–39). Similarly, exposure to NMs is found to be associated with several adverse outcomes such as impaired immune response, inflammation, fibrosis, emphysema, and tumor formation (40–45).

Additionally, EDCs have been reviewed to act as causative agents of metabolic disorders like type 2 diabetes mellitus (T2DM) (46, 47). The prevalence of T2DM has seen a tremendous global upsurge during the last few decades. A report from the International Diabetes Federation (IDF) estimated ~425 million adults (aged between 20 and 79 years) all over the world were living with diabetes in 2017 and predicted that by 2045 this will rise to 629 million. A large number of investigations to define the genetic and environmental bases of T2DM has been done to date but the definite reasons for a rapid increase in diabetes and obesity in the modern world largely remain unclear. Research in the arena increasingly indicates a major role played by environmental chemicals in diabetes and obesity, advocating that the environmental led metabolism disruption could form the “paradox of progress” (48). At present, only a few studies have examined the role of ENMs in the pathogenesis of T2DM. Chevalier and Fénichel reviewed both *in vivo* and *in vitro* experimental data along with epidemiological evidence to support an association of EDC exposure to the induction of insulin resistance and/or disruption of pancreatic β-cell function that leads to glucose homoeostasis related metabolic disorders (49). The evidence of ENM mediated alterations in glucose metabolism, insulin resistance and sensitivity, and homeostasis pathways are mostly indirect. The influence of EDENMs on the candidate genes of T2DM and their further impact on various molecular pathways are scarcely defined at present. Herein, we reviewed the recent literature that presented the effects of ENMs on molecular pathways involved in the development of T2DM. We also identified the knowledge gaps and challenges in the research area, which may provide directions for future research.

## An overview of the sources of human exposure to nanomaterials (NMs)

The probability of exposure to NMs in humans (50, 51) increases not only during production and application of ENMs, but also due to their emergence through several natural processes.

### Natural sources of NMs

We generally correlate exposure to NMs with human activities like the automobile industry, building construction and charcoal burning. However, 90% of the nano-particulate matter present in the environment is produced through natural phenomena such as dust storms, forest fires and volcanic eruptions, which significantly pollute natural resources, and affect human health. Dust storms are the main source of environmental NMs which can lead to respiratory issues, especially in subjects suffering from asthma and emphysema. Furthermore, dust rich in metal particles can lead to the generation of reactive oxygen species (ROS) (52), which may lead to an inflammatory response and influence pathogenesis of life style disorders such as T2DM and heart disorders.

### Anthropogenic sources of NMs

ENMs comprise the main category of anthropogenic release of NMs in the environment. These are produced and released into the environment by either intentional or unintentional human activities. The unintentional release of NMs occurs from the burning of natural fuels, wood, and wax (53–56). The intentional release occurs through the discharge of ENMs to rivers, landfills, soils and wastewater-treatment plants as well as from engineered products with embedded NMs. Intentional activities include the commercial synthesis of NMs, combustion of fossil fuel, manufacturing of NM embedded products, etc. Such products have found applications in biomedical, pharmaceutical, and agricultural domains. ENMs are rapidly being used in pharmaceuticals as carriers (57, 58) and as nano-formulations of drugs (59– 61) and for electro-analysis of pharmaceuticals (62, 63). ENMs also find diverse applications in agriculture (64) to increase the productivity by providing nano-scaled solutions primarily as pesticides (65), fertilizers (65), and biosensors (66). A fraction of these ENMs may also make their way into soil and water ecosystems, and therefore into drinking water and food products (67–69). Such observations have raised concerns regarding the presence of ENMs in consumer products and plausible association with human health and environmental degradation.

## Systems involved in uptake of ENMs into the human body

**Figure 2:**
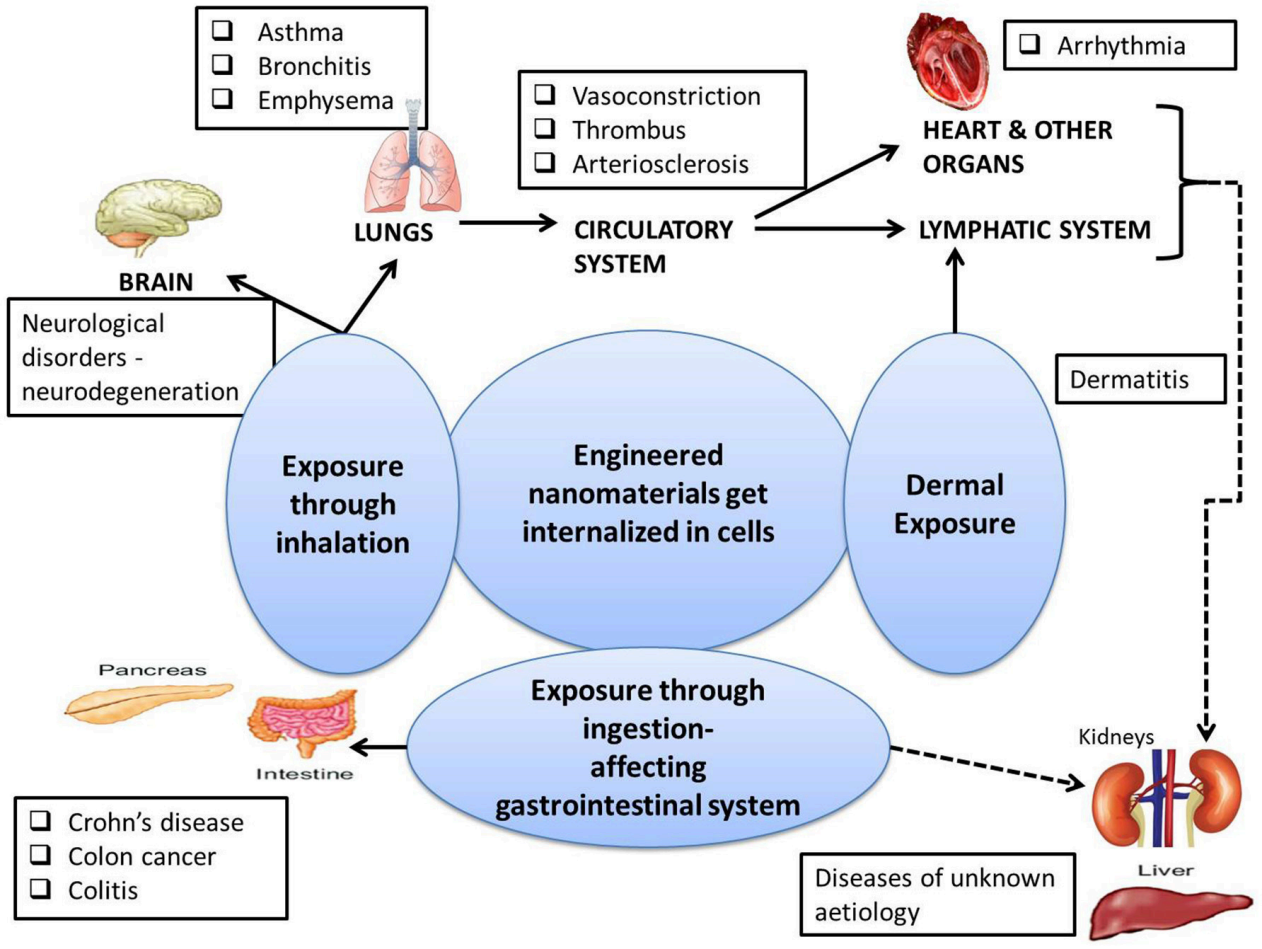
A concise overview of affected organs resulting in various pathological states due to the exposure to ENMs as described in the literature (42, 70). The directly and indirectly affected systems are shown by solid and dashed lines respectively.

Both natural and anthropogenic NMs enter the human body primarily through the respiratory system, skin and gastrointestinal (GI) tract, with further translocation to different tissues and organs as depicted in Figure 2 (71, 72). Considering the plethora of anthropogenic NMs, many ENMs may not be effectively removed and therefore can accumulate in different organ-systems over a period. Various cell types by which the ENMs are internalized include macrophages (73–75) endothelial cells (76), pulmonary epithelial cells (77–79), the gastrointestinal epithelium (80), blood cells (81), and neurons (82). Depending on their cellular concentration, the nanoscale particulate matter can mediate mutagenesis, damage to cell organelles, and eventually cell death.

### Respiratory system

The ENMs most prominently reach the body by inhalation and deposit throughout the entire respiratory tract (41, 70, 83). The soluble kinds of ENMs such as branched polyethylenimine and arginylglycylaspartic acid (RGD) based hydrophilic ENMs can be dissolved in the aqueous fluid lining the epithelium and can escape into the circulatory and lymphatic systems. However, the insoluble ENMs like nano-formulations of Au, Ag, Ti, Si, carbon etc., may accumulate in the lungs upon continued exposure, resulting in injury to the lung tissue (84). Recent research has demonstrated that inhalation of ENMs can deregulate the immune system and diminish the ability to fight infections (85). Also, as depicted in Figure 2, ENM exposure through the respiratory tract has been found to be associated with respiratory disorders, namely asthma (86), bronchitis (87, 88), and emphysema (89, 90); neuro-degenerative disorders, namely Alzheimer's (91), and Parkinson's (92, 93); and heart diseases (84).

### Skin

The extent of the uptake of ENMs by human skin is still debatable. The outer layer (stratum corneum) of the skin consists of a layer of dead cells and is generally impervious to materials having a pore diameter greater than micron size (94). However, many research studies show that NPs can penetrate the stratum corneum (95–97), especially when the skin is flexed. Dermal exposure of ENM has been reported to be associated with dermatitis (eczema) (98–100).

### Gastrointestinal system

ENMs present in food and cosmetics may enter the human body through ingestion by the gastrointestinal (GI) system. Nano-enabled applications, especially those for the food industry, dental care products and cosmetics, may lead to ENM exposure related toxicity (101–103) in humans. Major ENMs that are reported to cause cytotoxicity when ingested include nano-forms of gold, silver, and metal oxides of zinc, silica, and titanium (104–108). As also depicted in Figure 2, exposures to these NMs through the GI can lead to Crohn's disease, Colon disease and Gastroenteritis (109).

## Endocrine disrupting engineered nanomaterials (EDENMs) and mechanisms underlying the pathogenesis of type 2 diabetes mellitus (T2DM)

A global spike in the production of ENM has been observed in the twenty-first century, which has enhanced the exposure rate among workers and users. At present no occupational exposure level (OEL) for ENM has been defined and the assessment of exposure to ENM is challenging. Several research groups through various experimental models have demonstrated that ENM can elicit toxic responses that may be inferred from abnormalities in various organ-systems (110–113). The extent of toxicity conferred by ENM has been reported to depend upon their physicochemical properties including size, shape, chemical nature, and surface functionalization. The cytotoxic nature of ENM could eventually lead to cell death in a dose-and time-dependent manner (114). On the other hand, some other research studies that conducted short-term experiments on cultured cells and model organisms in order to evaluate the biocompatibility of ENM (115) suggested a low level of cytotoxicity of ENM. These studies advocated a huge potential for *in vivo* applications of ENM in the form of therapeutic and diagnostic reagents, although the effects related to a long-term exposure remain an unexplored domain at present. The studies on the long-term effects of ENM *in vivo* hold significant importance in designing and development of next-generation materials, but the mechanisms underlying ENM mediated toxicity in humans are less understood (116), Particularly, whether ENM exposure leads to endocrine-disruption and influences development of T2DM among the exposed subjects remains a topic of investigation.

**Figure 3:**
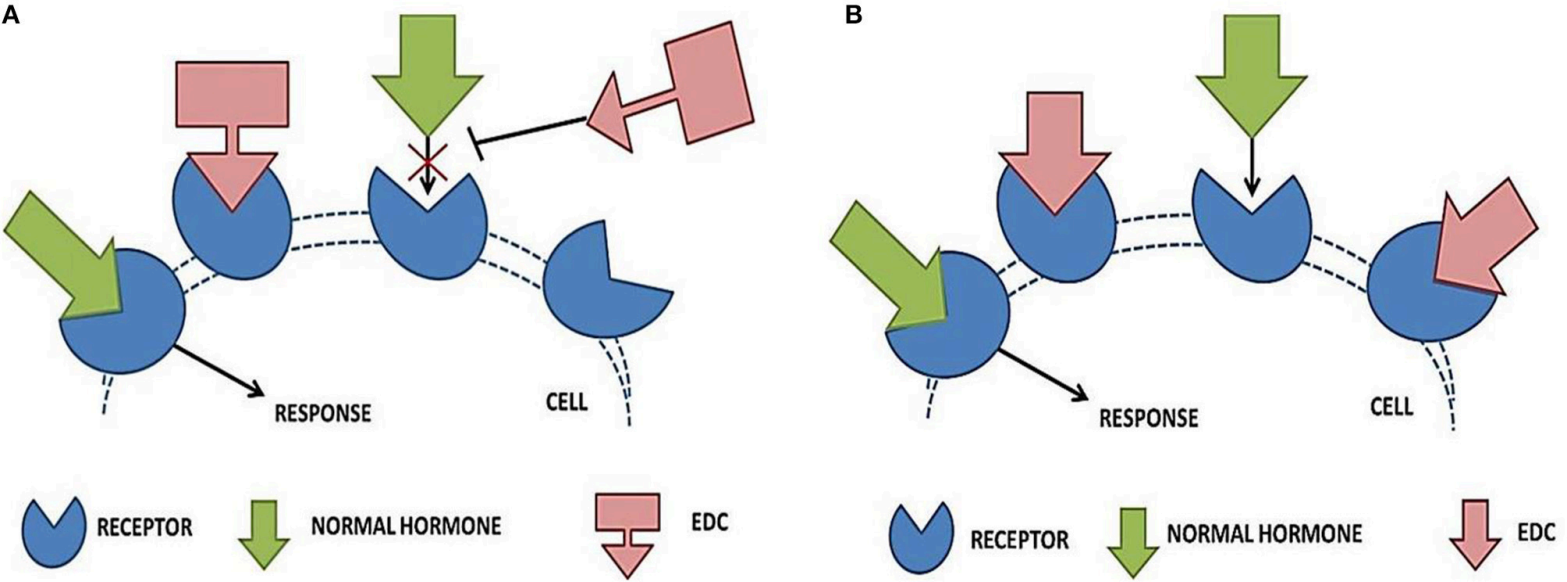
Effect of endocrine disrupting chemicals on receptor—hormone interaction. **(A)** Shows the blocking action of EDCs, where it binds to the receptor within the cell and blocks the endogenous hormone from binding. The normal signaling is blocked and the associated organ system (liver and pancreas) fails to respond properly. In **(B)**, the mimicking action of EDCs is depicted. EDCs completely or partially mimic the naturally occurring hormones, thereby interfering with the obvious physiological responses.

Contemporary *in vitro, in vivo* and epidemiological studies link human EDC exposure with obesity, T2DM and metabolic syndrome (47). Endocrine disruptors have a tendency to interact with the cellular receptors either by mimicking the natural hormones (Figure 3A) or by blocking the action of hormones (Figure 3B) (117).

Some studies have revealed the harmful effects of ENMs on endocrine functions, which suggests that ENMs may behave as potential endocrine disruptors, EDENMs (118–121). It is well acknowledged that endocrine disruption can often lead to the onset of metabolic disorders such as T2DM (122). Therefore, it is suggested that the ENM, which reportedly affects endocrine function, may induce T2DM. The EDENMs can behave in a similar fashion as the EDCs (13, 123, 124). The additional aspects of EDENMs over the traditional EDCs are small size, increased surface area and better uptake capability. Such properties of EDENMs enhance their chances of uptake and bioavailability, which further amplifies the deteriorating effect of the material on metabolic homeostasis (125–129) when compared to the other EDCs.

**Figure 4:**
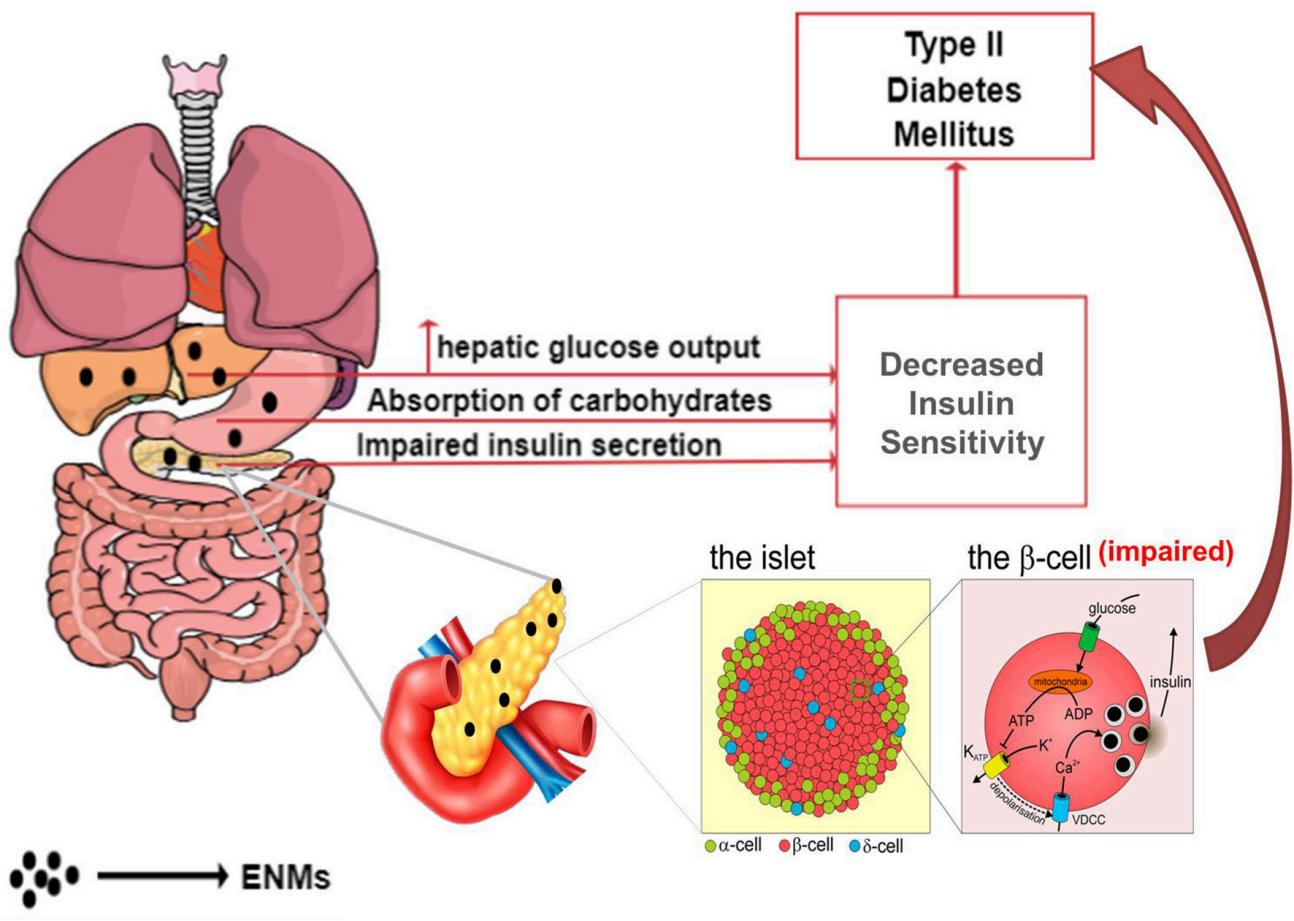
Effects of ENM on the gastrointestinal system, leading to the development of T2DM.

Working on similar ground as that of endocrine disruptors, ENMs may alter the normal metabolic state by affecting glucose homeostasis, leading to T2DM via the two chief mechanisms (Figure 4)—(i) decreasing insulin sensitivity and (ii) impairment of beta (β)-cells, resulting in a deleterious effect on insulin production (130–132).

### Reduced insulin sensitivity

Several factors lead to a reduction in cellular insulin sensitivity. Using the available *in vitro, in vivo* and epidemiological data, here we reviewed and illustrated the prominent mechanisms leading to decreased insulin sensitivity. The major focus in this review has been laid out—impairment of cellular insulin action, interaction with hormonal receptors, inflammation, and variations in homeostatic pathways. Each of these factors is explained in the following sections with evidence to show the deleterious effect of ENM on the pathogenesis of T2DM.

#### Impairment of cellular insulin action

Cellular metabolic reactions are often mediated by insulin via its action on the plasma membrane, intracellular enzymes and the nucleus. The cellular metabolism is regulated by various proteins (e.g., protein kinase C), receptors (e.g., receptor tyrosine kinase) or expression of secondary messengers (e.g., cyclic AMP, calcium, and diacylglycerol). These components control the translocation and activation of glucose transporter proteins (primary effects of insulin) (133). ENMs can influence the signaling mechanism by interfering with the normal actions of cellular messengers (130, 134–137).

**Figure 5:**
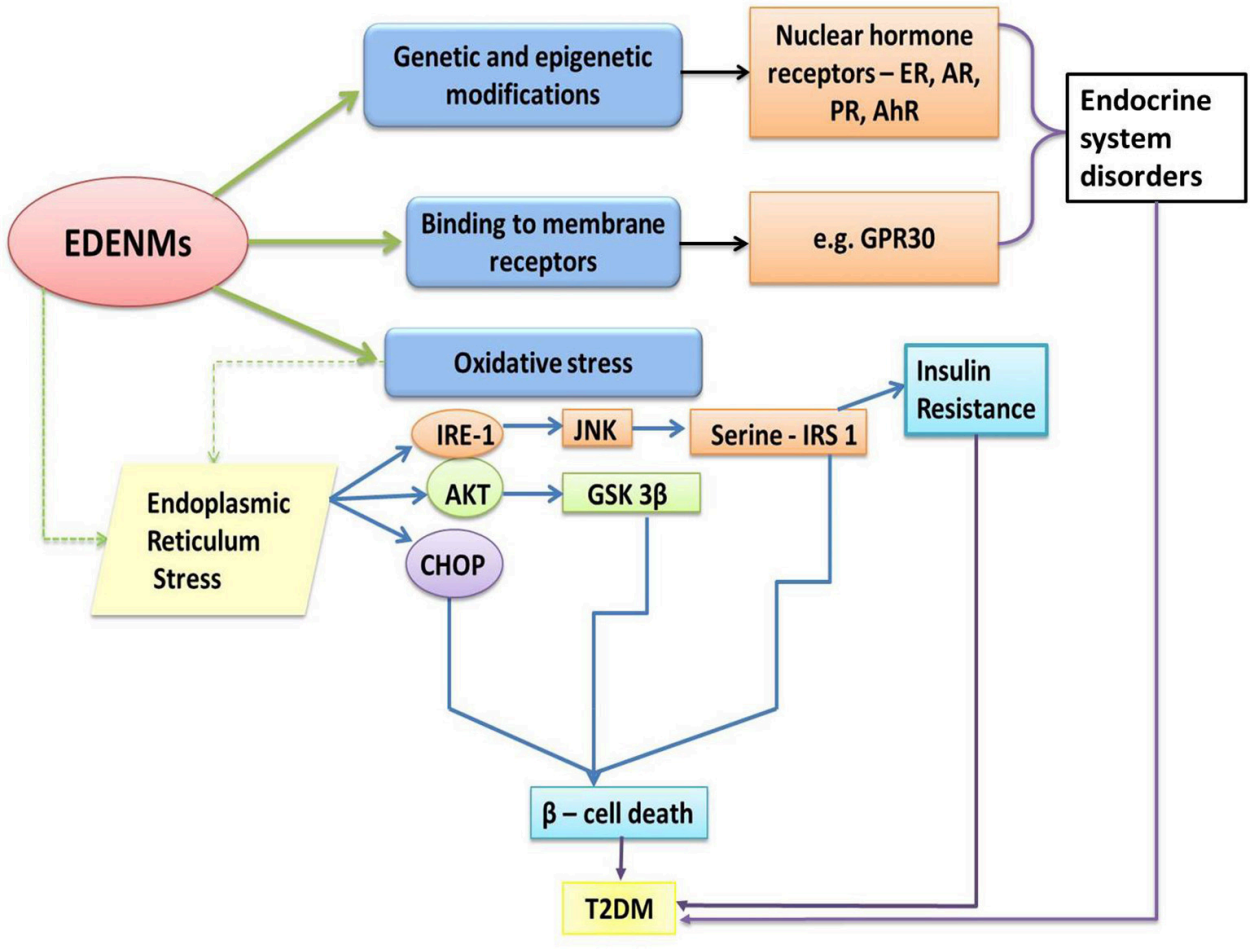
Molecular pathways influenced by EDENM, leading to the development of T2DM. (EDENM, endocrine disrupting engineered nanomaterials; T2 DM, Type 2 Diabetes Mellitus; ER, estrogenic receptor; AR, androgenic receptor; PR, progesterone receptor; AhR, aryl hydrocarbon receptor; GPR30, G-protein-coupled receptor for estrogen; IRE-1, inositol-requiring enzyme 1; AKT, alpha serine/threonine kinase; CHOP, CCAAT-enhancer-binding *protein* homologous *protein; JNK*, c-Jun N-terminal kinase; GSK 3β, Glycogen synthase kinase *3 beta; IRS-1*, Insulin receptor substrate 1). (The green solid arrows show the direct effect of EDENM. The green dashed arrow shows one of the major consequent events later resulting in T2DM, as further described by the blue solid arrows. Black solid arrows point toward the involvement of various receptors resulting in endocrine system disorders. Purple solid arrows infer the T2DM occurrence via various pathways).

**Figure 6:**
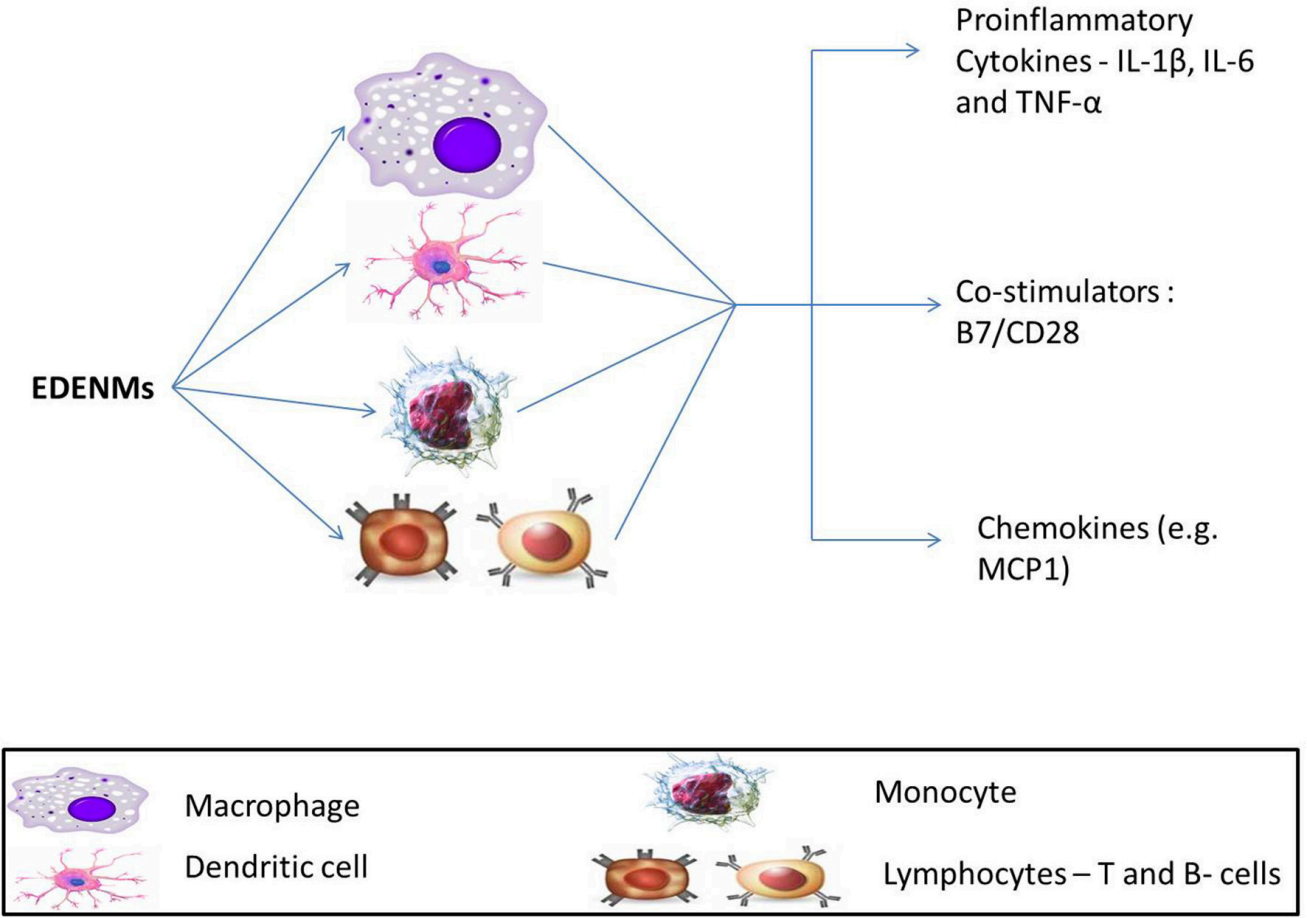
Effect of EDENM on various immune cells resulting in enhancement of expression of various pro-inflammatory cytokines (Interleukins, IL-1β, IL-6; TNF-α, Tumor Necrosis Factor-α), immunoglobulin superfamily members—B7/CD28 and chemokines (MCP-1, Monocyte chemoattractant protein-1).

The effect of titanium oxide nanoparticles (TiO_2_-NPs) on insulin resistance in liver-derived cells was evaluated in a research study (138). Briefly, the dose-dependent action of TiO_2_-NPs on Fao cells (rat hepatoma) was investigated. The cells were exposed to various concentrations (10, 50, 100, and 200 μg/mL) of TiO_2_-NPs. It was observed that treatment with 50–200 μg/mL of TiO_2_-NPs actuated insulin resistance by two mechanisms—directly affecting the hepatic cells to induce hepatic insulin resistance (Figure 2) and indirectly eliciting an inflammation response on macrophages (**Figure 6**) and hence releasing inflammatory cytokines such as Tumor Necrosis Factor –α (TNF-α) and Interleukins—1α/β (IL-1α/β). Administration of conditioned media form TiO_2_-NP-treated macrophages confirmed the activation of TNF-α, IL-6, IL-8, IL-1α, and IL-1β. These inflammatory cytokines caused insulin resistance in Fao cells. In addition, it was observed that direct exposure of TiO_2_–NPs to hepatic cells triggered the activation of stress kinases—c-Jun N-terminal Kinases (JNK) and p38, which attenuated the phosphorylation of Insulin Receptor Substrate (IRS) −1/2, and Glycogen Synthase Kinase (GSK) 3β, and led to abnormal insulin signaling (Figure 5).

An 8-week study was conducted to evaluate the effects of chromium nanoparticles (CrNano) on the hormone and immune responses of rats under heat stress conditions (118). Four groups (with 20 individuals per group) of Sprague–Dawley (SD) rats were randomly assigned to different dietary treatments. The first group was offered a basal diet as a control. The second, third, and fourth groups received a basal diet supplemented with 150, 300, and 450 μg/kg of CrNano, respectively. The treatment groups were then studied for various parameters governing overall metabolism. Measurement of sera concentrations of hormones and immunoglobulins with respect to the control group showed a decreased concentration of insulin and an increased concentration of insulin-like growth factor I and immunoglobulin G in the serum.

The same research group conducted another 6-week study to evaluate the effects of seven different levels of dietary CrNano (0, 75, 150, 300, 450, 600, and 1200 ppb Cr) in SD rats (139). Seven groups with 10 individuals per group of SD rats were randomly assigned to different dietary treatments. The results indicated that an addition of 300 and 450 ppb CrNano significantly decreased (*p*<*0.05*) the serum insulin level. It was observed that the Cr contents in the liver and kidney were significantly increased (*p*<*0.05*) by incremental dosage of CrNano from 150 to 1,200 ppb. The probable mechanism of reduction in peripheral insulin levels was ascribed to the activity of chromium in promoting hormone internalization into cells by increasing the membrane fluidity as explained by Evans and Bowman (140). This possibly led to altered insulin actions including binding of insulin to insulin receptors and the undesired interaction of insulin with various tissues such as adipose tissues and muscle tissues.

#### Interaction with estrogenic receptors

Estrogen receptors (ERs) expressed in adipose tissue, skeletal muscle, liver and pancreatic cells interact with estrogens and regulate metabolism. ER-α and ER-β play an important role in the regulation of glucose homeostasis by modulation of insulin sensitivity (141) and pancreatic insulin secretion (142). Additionally, in order to alter insulin secretion, the estrogen receptors facilitate the action of estrogen via G-protein coupled membrane receptors (137). The available reports suggest an antagonistic action of EDENMs toward the estrogenic receptors (127, 143, 144), which may lead to a decrease in insulin sensitivity and thereby alter glucose homeostasis.

The role of EDENMs in influencing estrogenic activity was demonstrated in a study where time-dependent (1, 3, or 5 h) treatment with 10 nm gold NPs to ovarian granulosa cells resulted in increased levels of estrogen (127). ERs have pathway modulation action via genomic and non-genomic approaches. While the genomic activity involves action of classical nuclear receptors to directly modulate the genes vital to homeostasis, the non-genomic route follows the use of kinases that activate signaling pathways resulting in the activation of ER pathway modulators. The increase in estrogen levels was suggested as indicative of the modulation of processes undergoing nuclear translocation, which might manipulate the normal gene expression for normal insulin signaling.

More evidence to support the endocrine-disrupting action of NMs was generated through illustrating the action of Cadmium Telluride Quantum Dots (CdTe QDs) (143). A dose-dependent study was carried out in uterine cells from female mice. The endocrine disrupting results were confirmed by a BrdU (BromodeoxyUridine) cell proliferation assay and a human ER 1 reporter assay for an assessment of ER activation.

#### Inflammation as the cause of insulin resistance

In line with the ongoing research on the effect of ENMs on the immune system, research observations have established that the secretion of inflammatory cytokines by various cell types (Figure 6) has an impact on insulin resistance (126, 128, 145–147). With their local and global action on different tissues, inflammatory cytokines have contributed to the development of insulin resistance and T2DM (146). The immune system recognizes ENMs as antigens and thus elicits an acute response. Under such circumstances, the adipose tissue upregulates the production of cytokines, which consequently leads to abrupt molecular signaling and the development of T2DM (148). Additionally, a substantial role of various ENMs in causing inflammation mediated toxicity has been previously reported (138, 149, 150).

Evidence from *in silico* experiments has provided insights into the putative mechanistic underlying the toxic response of carbon-based ENMs that causes T2DM. The effects of introducing C60 fullerenes and carbon nanotubes in a living system were modeled in a computational analysis (151). Results demonstrated that carbon NMs could be recognized as pathogens by the Toll-like receptors that may elicit innate immune response. Such a theory was well supported by expression of inflammatory secretory proteins like interleukin IL-8 and chemokine monocyte chemoattractant protein (MCP1). In another study, direct exposure of ENMs, including nano-diamonds and nano-platinum liquid, to human dendritic cells resulted in an enhanced expression of IL-1β, IL-6, and TNF-α. These cytokines are known mediators for an inflammatory response (152).

Metallic ENMs were also studied to assess any associated immune response by exploring a variety of mechanisms. Many research groups have conducted various experiments to understand the mechanistic for the inflammatory action of gold nanoparticles (GNPs) (125–127, 153). In one study, two different sized GNPs, 10 and 50nm, were chosen to look for the enhancement of gene expression for cytokines IL-1β, IL-6, and TNF-α by exposure to rat liver (146). The study also included experiments to understand the possibility of oxidative stress mediated damage to hepatic cells. The observations confirmed the generation of ROS in the presence of GNPs. These results clearly suggest that both the inflammation and oxidative stress related molecular pathways induced by ENMs play a significant role in the etiology of T2DM.

Another group investigated the combined effect of ZnO nanoparticle-mediated oxidative stress and immunogenic responses on immune cells. Immune cells, lymphocytes (activated memory and naïve) and monocytes, were assayed for the expression of (Interferon) IFN- γ, TNF- α, and IL-12 upon treatment with ZnO nanoparticles of varying sizes (4, 8, 13 and 20 nm). It was observed that activated memory lymphocytes were the most robust against ZnO exposure, followed by naïve lymphocytes. Monocytes were the most susceptible of the three choices. The effect on immune cells was size-dependent, with the smallest ZnO particles contributing most to the generation of ROS and toxicity (154). Such findings are in line with the previously discussed roles of inflammation and oxidative stress in the etiology of T2DM.

Evidence to support the involvement of metallic ENMs in eliciting immune response was put forward by creating a novel platform, MIMIC (Modular Immune *in vitro* Construct, an artificial system imitating the human immune system). This provides a digitalized platform where the response of the immune system to an antigen can be modeled computationally. Also, it is more flexible and faster as compared to traditional cell or animal models. The study was modeled in a predictive immunological construct for assessment of the effect of nano-TiO_2_ on the increase in the expression of IL-1β, IL-6, and TNF-α. The results demonstrated an enhancement in the secretion of pro-inflammatory cytokines on TiO_2_ exposure. Additionally, the expression of co-stimulatory B7/CD28 on dendritic cells was observed, which suggested a putative action of T-cells against TiO_2_ nanoparticles (145).

The available clinical-epidemiological research studies have clearly illustrated the involvement of abrupt immune action and ROS in the occurrence of T2DM. This evidence points toward a predictable association of EDENM-dependent disruption of immune activities and ROS generation that consequently leads to T2DM (155, 156).

Much evidence can be found in literature that establishes a link between the involvement of the immune system and diabetes bridged via inflammation. As substantial evidence, an epidemiological study was conducted to correlate inflammation (as monitored by variations in CRP level) and the development of diabetes (155). Another study evaluated the association between the release of inflammatory cytokines and blood glucose levels. A many-fold increase in cytokine concentration occurred during the low-grade systemic inflammation (157). It was suggested that the accelerated concentrations of interleukins and TNF-α may enable the migration of pro-inflammatory cytokines toward the insulin-signaling pathway. This may interfere with insulin signaling through phosphorylation of serine residues in insulin-receptor- substrate 1 (IRS 1) (158). Insulin-signaling may be blocked and insulin receptor (IR) stays dormant (159). In another epidemiological research, a diabetic Mexican-American population was studied for the effect of inflammation on the occurrence and prevalence of diabetes by testing for cytokines (IL-6, TNF-α, IL-1β, IL-8) and adipokine (adiponectin, resistin and leptin) levels in blood plasma. Increased plasma levels of these chemokines were found to be associated with increased blood glucose levels and T2DM (160). Several genetic association studies have suggested a role of polymorphism in the cytokine genes TNF-α and IL-6 in the development of diabetes and its associated comorbidities, retinopathy (161) and nephropathy (162, 163). Researchers have conducted a meta-analysis to probe into associations between −308 G>A (rs1800629) single nucleotide polymorphisms (SNP) in the TNF-α gene and T2DM (156). Another study reported significant association of genetic polymorphism in TNF-α, with an increased risk of T2DM in the Han Chinese population (138).

It is evident from the discussion that EDENMs may influence the onset of T2DM by triggering inflammatory pathways. Additionally, the formerly discussed arguments with epidemiological evidence presents a direct link between inflammation and T2DM. Such proof can be used to probe more into inflammation-dependent pathogenesis of T2DM due the exposure to ENMs.

#### Variations in homeostatic pathways

The homeostatic pathways involved in energy metabolism influences the overall development of an organism (136). In the case of metabolic disorders, the normal flow of homeostatic pathway is affected and the regulation is breached (136, 164). The EDENMs intervene with the factors related to insulin signaling. These factors include various kinases, including phosphatidylinositol 3-kinase (PI3K), protein kinase B, mammalian target of rapamycin (mTOR) and AMPK (AMP-activated protein kinase) which that tightly regulate sugar, lipid and amino acid homeostasis (164). This hampers the transduction of insulin signaling and results in diminished insulin action (165), which is followed by glucose metabolism malfunctions and development of T2DM.

*In vitro* studies have elucidated the action of metallic ENMs on homeostatic pathways. Findings from one study have established that heavy-metal NPs can elicit hyperglycemia (166). In another study, the influence of iron oxide (Fe_2_O_3_) NPs exposure on the signaling mechanisms was studied in murine hepatocytes (50). In this dose-dependent study, Fe_2_O_3_ NPs decreased the cell viability via the PI3K/Akt pathway. The exposure also resulted in a decrement of the intra-cellular antioxidant ability of hepatocytes. The study emphasized the role of exposure to Fe_2_O_3_ NPs (250 g/ml) in oxidative stress and apoptosis in the hepatic cells (167).

*in vivo* experiments have been carried out to explore the combined effect of ENM exposure and consequent abrupt functioning of oxidative stress and the homeostatic pathway on the endocrine system. Several *in vivo* studies have shown that the uptake of NMs has induced production of ROS (168). Physiological levels of ROS affected glucose metabolism pathways and insulin sensitivity (169). Moderate but long-standing oxidative stress has been found to be one of the major contributors in the onset of insulin resistance, and consequently T2DM (170). Oral introduction of TiO_2_ (anatase) nanoparticles to mice in a dose–dependent manner led to the accumulation of titanium in the liver, spleen, small intestine, kidney, and pancreas. Increased levels of titanium in these organs leads to insulin resistance, which was associated with increased phosphorylation of IRS1 (Ser307), JNK1, and p38 Mitogen activated protein kinase (MAPK), reduced phosphorylation of Akt (Ser473), and increased serum levels of TNF-α and IL-6 in the liver. An increase in the generation of ROS observed in the study also suggests a role for ENM in the induction of oxidative stress (171).

### Impairment of β cells and influence on insulin production

The low doses of endocrine disruptors can alter the functioning of the pancreas by affecting the physiology of both insulin- and glucagon-secretory cells, which can further disrupt the regulation of glucose and lipid metabolism. As depicted in Figure 5, loss in β-cell mass is predominantly governed by the pathways involved in oxidative stress and endoplasmic reticulum stress. It is reported that oxidative stress is accounted by the generation of excess ROS and contributes to T2DM through β-cell death (172). It has also been extensively reviewed that endoplasmic reticulum-stress plays a prominent role in causing apoptosis in pancreatic islets, resulting in β-cell death (173, 174).

A few metallic NMs have been shown to affect β-cells indirectly via influencing the kinases involved in transcriptional activation, followed by increased oxidative stress in the cells and finally apoptosis. In a research study carried out in macrophages, AuNPs of various sizes (4, 11, 19, 35, and 45 nm) suppressed NFκβ and JNK pathway activation. The process was observed due to de-methylation of CpG oligodeoxynucleotides (CpG-ODNs) motifs, making them act as immuno-stimulants (150). This effect was size dependent, with 4 nm AuNPs being the strongest suppressor. AuNPs potentially elicited an inflammatory response and induced oxidative stress as reviewed previously (128). Another study demonstrated the ROS generating capacity of Iron oxide (Fe_3_O_4)_ nanoparticles when H_2_O_2_ was used as the substrate (175). The hydroxyl free-radical generation reaction was biochemically similar to catalase and peroxidase action. It was inferred that the ROS may affect the pancreatic islet by JNK and NFκβ activation (176). Such observations supported the role of oxidative stress mediated inflammatory responses in β-cell damage, which may underlie the pathogenesis to T2DM.

Besides the above-discussed principle mechanisms, several unknown mechanisms underlying the development or pathogenesis of T2DM due to exposure to endocrine disrupters are also discussed in the available literature. The involvement of ultrafine particulate matter (key element of pollution) in the pathogenesis of T2DM is widely argued (52–56). Ultrafine particulate matter constitutes the ultrafine particles (UFPs), which are airborne particles with a thermodynamic/aerodynamic size of < 100 nm. Diesel engines as well as automobiles, along with sand dust, fires, hot volcanic lava, and ocean spray along with combustion activities such as the burning of biomass or wood, generate and then release UFPs into the air. The mechanisms underlying the development of T2DM due to exposure to air pollutants have not been completely deciphered to date, however, an epidemiological study has been done to see the effect of ambient UFPs and nitrogen dioxide in a cohort of Canadian-born residents (177). The results clearly indicated that exposure to UFPs led to increased risk of incident hypertension (hazard ratio = 1.03; 95% CI = 1.02, 1.04) and diabetes (hazard ratio = 1.06; 95% CI = 1.05, 1.08).

## Conclusions and perspectives

Evidence suggesting a role of EDENM exposure in the pathogenesis of T2DM is gradually emerging, but a comprehensive understanding of a wide range of nanomaterials and their effect on candidate gene pathways and other causal factors involved in T2DM remain to be completely deciphered. The fact that ENM can disrupt the endocrine system, which may eventually lead to T2DM, has gathered support from several *in vitro, in vivo*, and epidemiological studies. Altered glucose metabolism through various molecular mechanisms including insulin resistance, decreased insulin sensitivity, induction of oxidative stress pathways, and altered homeostasis have been reported by numerous laboratory studies that examined the effect of EDENM exposures. On the other hand, a few studies also report a contrasting effect of the same EDENM on T2DM related molecules. Many research groups have come up with a safer application of ENMs by illustrating their ability to work as therapeutics. Particularly, some of the biologically synthesized ENMs (178, 179) were found to possess therapeutic potential against T2DM (180–182). Other non-metallic and metallic NMs for similar applications are also reported (Table 1).

**Table 1:** Different categories of ENM used in therapeutics against T2DM.

**Type**	**Application**	**Study system**	**References**
**NON-METALLIC ENM**			
PEG-b-PLGA–biodegradable Polyethylene glycol and Poly (lactic-co-glycolic acid) copolymer (PEG-b-PLGA) Based cationic lipid-assisted nanoparticles (clans)	Anti-inflammatory action	Diet—induced type 2 diabetes mice	(183)
Chitosan	Gene delivery for Glucagon like peptide 1 (GLP-1), dipeptydil peptidase IV (DPP-IV resistant GLP-1 analogs) and siRNA targeting against DPP-IV	HT-29, HepG2, and Caco-2 cell lines	(184)
Insulin-loaded nano-carriers	Transdermal delivery of insulin	Streptozotocin-diabetic male Wistar rats	(185)
Alginic acid nanoparticles containing insulin	Sublingual delivery of insulin	Streptozotocin-induced diabetic male Wistar rats	(186)
Insulin-containing Polyethylene imine-based nanoparticles	Insulin–delivery	Sprague Dawley rats	(187)
PLGA as the carrier to prepare recombinant human epidermal growth factor (rhEGF) nanoparticles	Diabetic wound healing	Diabetic rats	(188)
Insulin encapsulated in polyalkylcyanoacrylate nanocapsules	Hypoglycemia effect	Diabetic rats	(189)
Nanoparticles from dextran, poly (α-1,6 glucose), physically cross-linked with the tetra functional glucose-binding protein, Con A	Controlled delivery of insulin	*In vitro* studies	(190)
Injectable insulin nano-particles	Insulin delivery	Subcutaneous administration in diabetic mice	(191)
**BIOSYNTHESIZED ENM**			
*Eysenhardtia polystachya*-loaded silver nanoparticles (EP/AgNPs)	Antidiabetic activity	Pancreatic β cells, INS-1 cells, and *Danio rerio*	(180)
Gold nanoparticles (AuNPs) synthesized using *Gymnema sylvestre R. Br* Plant extract	Antidiabetic activity	Wistar albino rats	(182)
Gold nanoparticles (AuNPs) synthesized using *Cassia auriculata* plant extract	Increasing plasma insulin activity	Alloxan induced albino rats	(181)
**METALLIC ENM**			
Insulin-coated gold nanoparticles (INS-GNPs)	For controlled and prolonged glucose regulation was reported	Intravenous and subcutaneous administration to diabetic mouse model	(192)
Gold NPs and aspartic acid-capped gold nanoparticles	Insulin delivery	Diabetic Wistar rats	(193)
Gold nanoparticles and Dextran–insulin conjugates	Insulin delivery	Mouse 3T3-L1 cell line	(194)
Mesoporous silica nanoparticles (MSNs)	Gluconic acid-modified insulin (G-Ins) proteins labeled with fluorescein isothiocyanate (FITC-G-Ins) were immobilized on the exterior surface of MSN which served as caps to encapsulate cAMP molecules inside the mesopores of MSN	RIN-5F	(194, 195)
Selenium nanoparticles (SeNPs)	Oral delivery of insulin to enhance the antidiabetic effect	Normal (Sprague-Dawley, SD) and type II DM (Goto-Kakizaki, GK) rats	(196)

In an interesting study, an increase in cell viability, ATP/ADP ratio and secretion of insulin in response to glucose stimuli in the isolated pancreatic islets when treated with metallic nanoparticles prepared from cerium oxide (CeO_2_-NPs) at a concentration of 100 nmol/L, either alone or in combination with 30 nmol/L sodium selenite, was reported (197). These findings could possibly be ascribed to the anti-oxidant potential of CeO_2_-NPs, which may exert a different effect on the insulin release. In a similar study, the effect of zinc oxide nanoparticles (ZON) on oxidative stress-mediated pancreatic β-cell death was investigated in rats (RIN5f). The cellular levels of antioxidant factors and the rate of apoptosis were assessed in correlation with ZON uptake. RIN5f cell treatment with ZON (30 and 100 μg/ml) resulted in cytotoxicity, oxidative stress and apoptosis. In contrast, the sub-cytotoxic concentrations (1, 3, 10 μg/ml) protected RIN5f cells from hydrogen peroxide (H_2_O_2_)-induced oxidative stress by the reducing the cellular levels of ROS, increased SOD activity and GSH, and reduced cell death (198). The findings reported in the above-mentioned studies indicated that nanomaterial-type and size dependencies on the associated cellular toxicity are not precisely explored. An incomplete characterization of ENM may underlie discrepancies observed in available scientific reports. Confusion created from such incomplete explorations demands for the development of a common understanding in the area of ENM characteristics (shape, size, surface area, mass concentration, or a combination of these), which should be a prerequisite to toxic effect determination. This broadens the scope of research to particularly define physicochemical properties of ENM for their sustainable bio-medical applications. Considering this, ENMs for vital bio-medical, pharmaceutical, agricultural, and environmental applications are required to be well characterized for their uptake to various cell/ tissue types, interaction with cellular organelles and cell mechanistic aspects within the intracellular environment (199).

Since *in vitro* systems do not necessarily mimic the human system, results obtained from such experiments need to be replicated through *in vivo* studies conducted in different model animals. Additionally, *in vivo* studies that address potential effects of EDENM on the development of T2DM at a large scale such as a population, a community, or an ecosystem with sufficient power are necessarily required. Further, most of the *in vivo* studies have been conducted on small rodents, specifically rats, mice and hamsters, which may not be optimum for studying the long-term toxic effects of nanomaterial and makes it difficult to extrapolate the observed results to humans. Experiments by including other model systems, which are more closely related to human systems, like *Danio rerio, Daphnia magna*, and *Caenorhabditis elegans* need to be conducted in more numbers. Furthermore, pre-clinical *in vitro* studies, such as those using blood samples from a control population, can also be conducted for impact-assessment of EDENMs (200). Additional support to the candidate mechanisms underlying EDENM mediated T2DM and identification of novel pathways can be achieved through the application of the “-omics” approach, which at present is virtually lacking. Also, developing high-throughput pre-screening (HTPS) and quantitative structure-activity relationship (QSAR) methods for *in silico* screening and prediction would be extremely important to comprehend the effect of EDENM (201).

We emphasize the importance of research on safety aspect of ENMs, which would minimize the uncertainties regarding the health and environmental issues surrounding these advance-materials and help in the development of safe applications of nanotechnologies.

## Author contributions

PS was the Principal Scientist, involved in conceptualization of the review, study design, data analyses, data compilation, manuscript writing, critical inputs, and finalization of the manuscript. AP contributed toward data analyses, data compilation, manuscript writing, critical inputs, and finalization of the manuscript. SG was involved in data compilation and manuscript writing. All authors have read and approved the final manuscript.

### Conflict of interest statement

The authors declare that the research was conducted in the absence of any commercial or financial relationships that could be construed as a potential conflict of interest.

## Abbreviations

AgNPs: Silver Nanoparticles
Ahr: Aryl Hydrocarbon Receptor
AKT: Alpha Serine/Threonine Kinase
ALS: Alloxan Sensitive
AMPK: AMP Activated Protein Kinase
AR: Androgenic Receptor
AuNPs: Gold Nanoparticles
B7/CD28: Immunoglobulin Superfamily Members
BrdU: Bromodeoxyuridine
CARDIA: Coronary Artery Risk Development In Young Adults
CdTe Qds: Cadmium Telluride Quantum Dots
CeO_2_-NPs: Cerium Oxide Nanoparticles
CHOP: CCAAT-Enhancer-Binding Protein Homologous Protein
Cpg-Odns: Cpg Oligodeoxynucleotides
CrNano: Chromium Nanoparticles
CRP: C-Reactive Protein
EDCs: Endocrine-Disrupting Chemicals
EDENM: Endocrine-Disrupting ENM
ENM: Engineered Nanomaterial
ER: Estrogen Receptors
G6PDH: Glucose-6-Phosphate Dehydrogenase
GI: Gastrointestinal tract
GK: Goto-Kakizaki
GLUT: Glucose Transporter
GNPs: Gold Nanoparticles
GPR30: G-Protein-Coupled Receptor For Estrogen
GPX: Glutathione Peroxidase
GR: Glutathione Reductase
GSH: Glutathione
GSK: Glycogen Synthase Kinase
H_2_O_2_: Hydrogen Peroxide
HIP: Human Amyloid Polypeptide
IFG: Impaired Fasting Glucose
IFN: Interferon
IL: Interleukin
INS-GNPs: Insulin-Coated Gold Nanoparticles
IONPs: Iron Oxide (Fe_2_O_3_) Nanoparticles
IPCS: International Programme On Chemical Safety
IR: Insulin Receptor
IRE-1: Inositol-Requiring Enzyme 1
IRS: Insulin Receptor Substrate
JNK: C-Jun N-Terminal Kinases
LDL: Low Density Lipoproteins
MCP1: Monocyte Chemoattractant Protein 1
MDA: Malondialdehyde
MIMIC: Modular Immune *in vitro* Construct An Artificial System Imitating The Human Immune System
MSNs: Mesoporous Silica Nanoparticles
Mtor: Mammalian Target of Rapamycin
NMs: Nanomaterials
NPs: Nanoparticles
PEG-B-PLGA: Biodegradable Polyethylene Glycol And Poly (Lactic-Co-Glycolic Acid) Copolymer
PI3K: Phosphatidylinositol 3-Kinase
PPAR: Peroxisome Proliferator Activated Receptor
PR: Progesterone Receptor
PTP: Phosphotyrosine Phosphotase
rHEGF: Recombinant Human Epidermal Growth Factor
ROS: Reactive Oxygen Species
SD: Sprague–Dawley
SeNPs: Selenium Nanoparticles
SNP: Single Nucleotide Polymorphisms
SOD: Superoxide Dismutase
T2DM: Type 2 Diabetes Mellitus
TiO_2_-NPs: Titanium Oxide Nanoparticles
TNF-α: Tumor Necrosis Factor –A
UFPs: Ultrafine Particles
WHO: World Health Organization
ZON: Zinc Oxide Nanoparticles
β-Cells: Beta Cells.
